# Investigating biocomplexity through the agent-based paradigm

**DOI:** 10.1093/bib/bbt077

**Published:** 2013-11-12

**Authors:** Himanshu Kaul, Yiannis Ventikos

**Keywords:** agent-based model, biological complexity, computational modeling, cell, emergence, hybrid models

## Abstract

Capturing the dynamism that pervades biological systems requires a computational approach that can accommodate both the continuous features of the system environment as well as the flexible and heterogeneous nature of component interactions. This presents a serious challenge for the more traditional mathematical approaches that assume component homogeneity to relate system observables using mathematical equations. While the homogeneity condition does not lead to loss of accuracy while simulating various continua, it fails to offer detailed solutions when applied to systems with dynamically interacting heterogeneous components. As the functionality and architecture of most biological systems is a product of multi-faceted individual interactions at the sub-system level, continuum models rarely offer much beyond qualitative similarity. Agent-based modelling is a class of algorithmic computational approaches that rely on interactions between Turing-complete finite-state machines—or agents—to simulate, from the bottom-up, macroscopic properties of a system. In recognizing the heterogeneity condition, they offer suitable ontologies to the system components being modelled, thereby succeeding where their continuum counterparts tend to struggle. Furthermore, being inherently hierarchical, they are quite amenable to coupling with other computational paradigms. The integration of any agent-based framework with continuum models is arguably the most elegant and precise way of representing biological systems. Although in its nascence, agent-based modelling has been utilized to model biological complexity across a broad range of biological scales (from cells to societies). In this article, we explore the reasons that make agent-based modelling the most precise approach to model biological systems that tend to be non-linear and complex.

## INTRODUCTION

Quantifying, and even defining, the complexity paradigm has been challenging due to differences among systems that are considered complex in terms of their information content, dimensionality and basic functional units [1]. However, complex systems are usually characterized by the presence of numerous (sometimes) heterogeneous components that can interact non-linearly to yield a large variety of possible configurations [1], absence of rigid boundaries [2], flexibility in terms of component membership (components can have multiple memberships) [2], and the ability to display emergent, self-organizing and adaptive behaviour [3]. Although complicated systems (such as the nerve network found in sea slugs of the genus *Aplysia* [4])—relatively straightforward to define in mathematical terms—partially share the first characteristic of a complex system (i.e. it may possess numerous interacting components), they differ from complex systems in terms of connectivity among system components.

A complicated system may have numerous components, but it operates linearly. In other words, it operates in order (elements are not connected, and hence there is an absence of dynamics between them [3]), as opposed to chaos where every element is connected to every other element [3]. Complicated systems are therefore predictable [4]. Complex systems on the other hand operate at an intermediate state between order and chaos [2–4]. Their outcomes, therefore, are more difficult to predict. This lack of predictability results from the diversity of interactions that the heterogeneous components are capable of engaging in. As a result, simulating the spatiotemporal evolution of complex systems requires identification of the nature of functional/hierarchical relationship(s) between the interacting components. A special case of the complex phenomenon, and the subject of this review, is biocomplexity.

Biocomplexity, as proposed by the US National Science Foundation [5], emerges from ‘dynamic interactions among the biological, physical, and social components of the Earth’s diverse environmental systems’, and, as the term itself suggests, is a property of biological systems. According to Michener *et al*. [6], it arises when temporal, conceptual and spatial boundaries of a biological system are breached. Appearance of emergent behaviour (whether evolutionary, self-organizing or adaptive) is the corresponding result. A philosophical [7] and computational [8] discussion on emergence can be found in the indicated references. Metabolic pathways, differentiation, tissue morphogenesis, embryogenesis and ecosystems are but a few examples of complex biological processes, from the sub-molecular to the planetary.

Complexity in biological systems is a result of the bi-directional cross-talk that exists among system components, and between these components and their (micro)environments, which is further augmented by the heterogeneity of system components. Furthermore, the macroscopic behaviour in biological systems is underpinned by a vast range of interactions between interconnected parts at a multitude of scales in the absence of a central organizing structure [9,10]. Investigating the governing dynamics that regulate such systems entails experimentation of the trial-and-error flavour, but the, sometimes, colossal gaps between system outputs under slightly varying initial conditions have done little to decrypt the black-box-like modus-operandi of such systems. As such, there has been, until recently, an inconvenient absence of computational models capable of making accurate quantifiable predictions in the literature. To understand precisely the nature of biological systems, therefore, requires supplementing the empirical with the quantifiable, and hence, a synergistic collaboration of experimental and computational methods.

Computational tools utilized to model biological phenomena can be categorized, broadly, as continuum or discrete. Whereas the former describe the numerical changes of the variables that represent the system, the latter can indicate how and why the dynamics involving system components operate [11]. Continuum approaches, such as those used to address traditionally the governing laws of fluid or solid dynamics, employ classical differential equation-based models that may have numerical or approximate solutions. However, mathematical equations representing either a collection of cells or organisms or their (micro)environment do not lend themselves as the most precise form of ontologies for biological systems. While the continuum approach has been applied successfully to predict macroscopic observables such as regional cell numbers, traction forces and wound morphometry, to name a few, classic continua are not dynamic—unlike biological systems they do not change their material properties over time [12] (although, of course, numerous continuum computational methods that attempt to incorporate such variations have been proposed). Their shortcoming is even more pronounced when simulating emergent behaviour that ‘arises through “self-organization” and that could not have otherwise been characterized a priori’ [9]—an event that is probably beyond the remit of the continuum approach.

Discrete approaches, such as cellular automata [13,14] (CA)—which employ interacting finite-state machines [15]—or the cellular Potts modelling approach (CPM) [16]—which simulates systems by mapping cells to domains on a lattice—can capture (i) the non-homogeneous character of biological systems (which is also responsible for their complexity) and (ii) the emergence of global patterns from underlying rules, in a manner more faithful to cellular systems than their continuum counterparts. Agent-based modelling (ABM) is one such discrete approach. The rest of the article is devoted to, among others, an introduction to ABM, definitions of the term agent, discussion on the agent-based philosophy and its ontological relevance to biological systems, a brief discussion on the strengths and weaknesses of the two computational approaches, a generic template for creating models using the agent-based paradigm and reviewing agent-based models, of either standalone (purely discrete) or hybrid (continuum–discrete) variety, that have been employed to simulate biological phenomena across a broad range of biological scales.

## AGENT-BASED MODELLING

To model biological behaviour, investigators have applied a host of computational approaches, such as casting laws in partial differential equations form, gas kinetic theory, CA, Brownian agents, Bayesian networks, co-clustering latent variable models, etc. [17–22], that have had their fair share of success in predicting certain stem cell behaviour, given the model assumptions and boundary conditions. Yet, models of such flavour face challenges when attempting to offer adequately comprehensive insights into the processes that govern the behaviour of biological systems [23]. Furthermore, global observations in continuum models represent averaged values [24] and assume homogeneity of system components. In doing so, the continuum models run the risk of ignoring a system’s low-level details [14,25], a feature quite central to biological function [26,27].

An agent-based approach is typically utilized when (i) individuals to be modelled are locally interacting discrete entities that display adaptive behaviour [28], (ii) the population comprising the discrete entities is heterogeneous [24], (iii) the topology of interactions itself is heterogeneous [24], (iv) spatial considerations are important because spatial localization of individual entities takes precedence [24,29], (v) emergent phenomena are the primary interest [24,29] and (vi) the number of individuals to be modelled is relatively small (generally less than a billion [29]). Biological systems therefore fall under the remit of ABM. As such, assuming that the aforementioned criteria are met, agent-based models can be employed to model any scale of interest (from cells to societies). ABM ‘discretizes’ the system being modelled into a collection of autonomous decision-making entities that act at each of several discrete time steps based on their local information and rule-set attributed to them [9,24,30,31]. Agent-based models are, therefore, typically composed of agents (autonomous entities), rules (logic or mathematical), a simulation environment (source of local information) and initial and boundary conditions [14,32]. An advantage that ABM offers over other computational approaches is its ability to model global emergent phenomena through the rule-set assigned at the agent level [19] alone [31].

Based on the set of rules, local information and boundary conditions, agents interact with each other and their environment, thereby transitioning, asynchronously, between a finite number of states. The states can be recorded, monitored and accessed at any moment to exhibit the evolution of an agent or a set of agents over the duration of the simulation [32]. Furthermore, repetitive-competitive interactions between agents are a feature of ABM [24]. While both ABM and CA employ interacting finite-state machines and are, therefore, quite similar in terms of their implementation, there are differences that make the agent-based approach a more convenient option. ABM relies on agents that are mobile, as opposed to the static grids in ‘classical’ CA [9]. ABM is characterized by asynchronous agent behaviour, which means that agents can update their states independent of one another [9,14]. Furthermore, ABM allows incorporation of stochastic elements in the rule-set attributed to the agents [14]. More importantly, CA lacks—in most implementations—internal memory, a feature of agents (discussed in the next section). This leads to a combinatorial explosion of states when considering even simple communication using CA [14]. As a result, when it comes down to representing non-trivial complex systems, the CA approach performs sub-optimally [14]. And, finally, the ease with which stochastic elements can be introduced in the agent-based rule-sets [33] makes it more consistent with the operation of biological systems compared with the deterministic rule-sets [34] of the classic CA.

## DEFINING AN AGENT

Introduced by Laycock [35], an agent (or a stream X-machine [35]) is a Turing-complete finite-state machine [29] (computational system that can simulate any single-taped Turing Machine [36]) that contains a finite set of internal states, a set of transition functions operating between states, an internal memory set and a language for interacting with other agents (XMML) [37]. According to Jennings [38], agents are the new theoretical model of computation, which reflect current reality more closely than Turing machines [36].

From a qualitative perspective, as defined by Wooldridge [39], ‘an agent is an encapsulated computer system that is situated in some environment and that is capable of flexible, autonomous action in that environment in order to meet its design objectives’. Therefore, by definition, an agent possesses well-defined boundaries and interfaces, has the ability to sense its environment (and act on its environment), can control its internal state as well as behaviour, has particular goals to achieve, can act in the anticipation of future goals and responds in timely fashion to changes that affect its environment [38].

**Figure 1:**
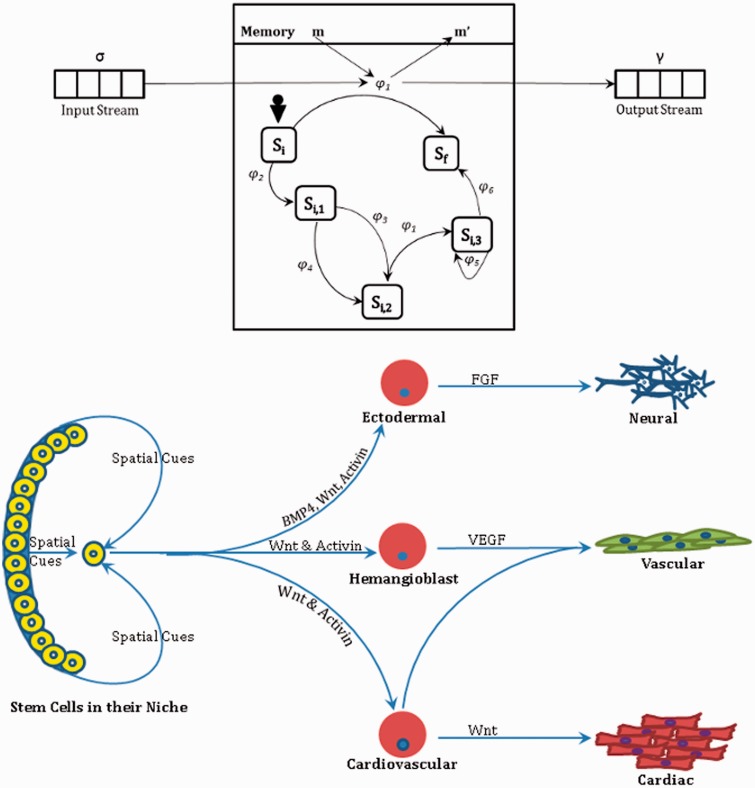
This figure highlights the parallels between an agent and a cell. *Top*: A communicating agent (stream X-machine); adapted from [29]. *Bottom*: Cell decision-making; signalling cues derived from [40]. Depending on the multitude of input signals that a cell responds to, it transitions into a phenotype based on hitherto unknown biological rules. The input signals (represented on the arrows) can be spatial, chemical or electrical and induce a response from the cell. The cell in its new transition state seems to be quite aware of its latest phenotype, a feature that in the ABM is represented by the update of agent-memory.

**Figure 2:**
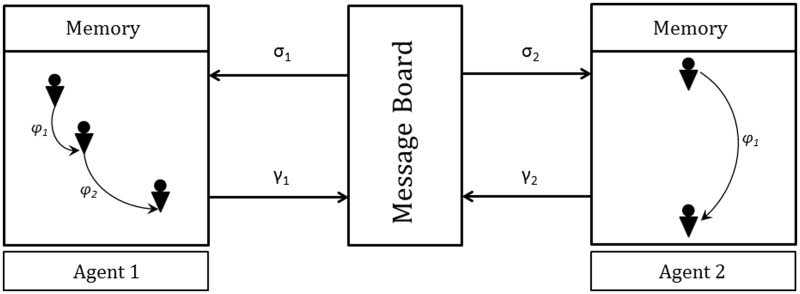
The dynamics of X-machine communication. The message board maintains a database of all the messages sent by the agents. The agents read, and send, messages from (and to) the message board. Adapted from the FLAME user manual available at http://www.flame.ac.uk/docs/.

An agent *X* can be represented quantitatively as [13]:

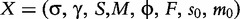

where σ is the set of input, *γ* is the set of output, *S* denotes the set of states, *m* denotes the variables in the memory, *Φ* denotes the set of partial functions *φ* that map an input and memory variable to an output and a change on the memory variable (*φ*: *σ* × *M* → *γ* × *M*), *F* is the next state transition function: *F*: *S* × *φ*, *s_0_* is the initial state and *m_0_* is the initial memory. A common message board to (and from) which messages are posted (and read) assists the agents in communicating with each other. Figure 1 shows an X-Machine agent and Figure 2 represents communication between two X-machines.

**Figure 3:**
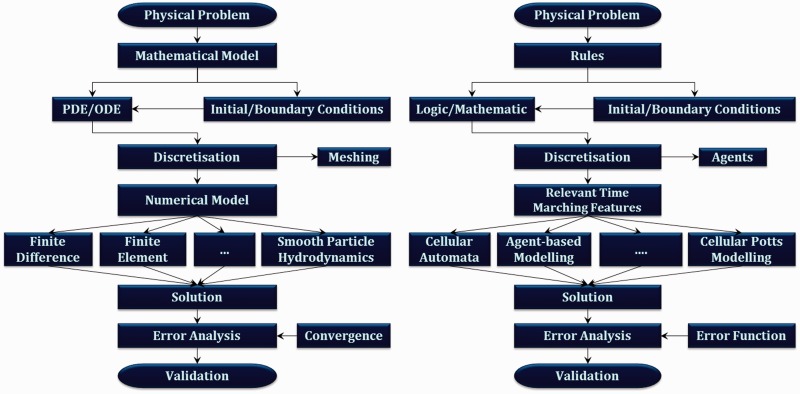
The figure shows the parallel between how the continuum and discrete approaches are used to simulate biological phenomena. Calculating the error function in ABM is analogous to setting the convergence criteria in continuum methods. Similarly, meshing a geometry to assign discrete locations where the differential equations are solved is equivalent to distributing agents (in the environment) capable of transitioning between a finite set of states based on the logic/mathematical rules assigned to them.

The state transition functions (*φ*) respond to events considering both the environmental input *σ* as well as the current internal state. For example, a communicating X-machine with an initial state *i* and an initial memory *m* on receiving input *σ*, depending on *σ* and *m*, will/may(/will not) change its state producing an output γ and updating the memory to *m*'. This modelling mechanism provides a sensible way of dealing with problems associated with state explosion, which afflict many efforts at modelling complex biological systems [9,29]. Also, being inherently hierarchical, an X-machine is able to link different modelling paradigms [29].

## THE AGENT-BASED PARADIGM AND BIOLOGICAL COMPLEXITY

What makes ABM a well-suited approach to study complex systems? Booch [38,41] identifies three tools that are required to analyse complexity: decomposition, the ability to break down a complex system into smaller, more manageable, chunks that can be dealt with in relative isolation; abstraction, the process of defining a simplified model that can explain the salient features of a system (at the expense of less relevant detail) and organization, the process of managing and identifying the interrelationships between the problem-solving parts [38]. ABM satisfies all three requirements completely.

A natural way of representing a biological system is (as discussed later) to decentralize the control or introduce multiple loci of control [24,38]. This is an intuitive way of representing biological systems, as decision-making is limited to the agent’s local situation rather than some external entity’s perception of the situation [38]. ABM achieves that by decomposing the problem in terms of entities that engage in flexible high-level interactions [9,24,28,38]. A significant benefit of the flexible nature of agent interaction is that the agent decision-making regarding the nature and scope of its interactions can occur at run time. This allows the user to bypass the need to specify every possible inter-agent link [38] (an impossibility given the nature of the systems’ complexity).

The fact that the agent-oriented mind-set provides suitable abstractions is evident from the availability of the rich set of structures that are employed to represent and manage organizational relationships [38,42–44] and interaction protocols that overlook the formation of new groupings and disbanding of existing ones [38,44,45]. The fact that collectives, such as teams, can be modelled [38] as well further supports the aforementioned claim. Finally, the ability of the paradigm to conduct organizational updating during run time (in case of an agent being destroyed or differentiating into a phenotype of profoundly different nature, for example) [38] makes an ineluctable case in favour of the agent-based philosophy as the most suitable for dealing with organizational relationships appropriate for complex systems.

But why consider the agent-based approach to simulate biological phenomena? The answer lies in the interaction-reliant methodology of the agent-oriented approach, seemingly consistent with the mode of operation of biological systems. The macroscopic behaviour in the biological world is underpinned by a whole range of interactions across all scales (of course). For example, in the tissue context, it is cellular interactions—between themselves as well as their microenvironment (fibres, for example)—that play a central and pivotal role in determining the functionality and architecture of the evolving system. While there are a host of complex intracellular processes that regulate cellular behaviour, a cell can be assumed, before all other intracellular components, as the autonomous entity [46]. Therefore, the argument boils down to (in the tissue context) whether an agent can serve as a suitable ontology for the cell. We shall show that a parallel can be easily drawn between the two (refer to Figure 1).

As mentioned previously, an agent (i) possesses well-defined boundaries, (ii) has the ability to sense its environment and act on its environment, (iii) can control its internal state as well as behaviour, (iv) has particular goals to achieve, (v) can act in the anticipation of future goals and (vi) responds in timely fashion to changes that affect its environment. Cells can be easily visualized as ‘agents’ in the light of an agent’s aforementioned properties. After all, cells are in fact embedded in an environment; possess boundaries; maintain a dynamic bidirectional cross-talk with their environment [22,47–54], thus being acted by and acting on their environment; have the ability to control their behaviour through secretion of relevant autocrines and act in anticipation of future goals as a result of metabolic sensing (when cells try to gauge the ‘needs’ of a tissue, as suggested by Scadden [54]) or signalling (as occurs in a functional immune system [55,56]). This analogy can be easily extended to tissues, organs, organisms and colonies, in equal measure.

The greatest advantage offered by the ABM to the user, however, is its ability to capture emergent phenomena [19,24,29–31,38,57–59], which the continuum approach finds more challenging to describe. Furthermore, self-organizing systems formed by cells, where individual cells react to their environment and to each other [58,59], are examples of emergent phenomena observed in biological systems. It is the local interactions between cells that determine the architectural and functional features of the entire system [58]. Multi-agent systems, a variant of agent-based models, fit nicely into this problem and with their application it has become possible to understand the self-organization behaviour of stem cells and deal with their emergent global behaviour [58].

The decentralized manner of targeting complex systems is perhaps the strongest argument in favour of using ABM to simulate biological processes. Unlike the continuum approach with a distinct cause-and-effect motif, the agent-based approach relies on interactions—among agents coupled with their environment—to capture and explain macroscopic observables. Biological systems, as mentioned previously, thrive on such interactions and there seems to be an absence of a strict causative impulse within them. The flocking of birds [60,61], the aggregation displayed by a slime mould colony under duress [62], the outcome of cellular phenotype based on the microenvironment [63] and the multiple streams of consciousness [64] that seem to govern human endeavours, all seem to have little centralized control [61], if any. On the contrary, it is the underlying agent-like behaviour that gives rise to each of the aforementioned global observables. The flocking behaviour is borne out of each bird in the flock responding to the movements and positions of its neighbouring birds (an example of a lower-level rule) [65], the queen termite/ant has little centralized role to play in orchestrating the dynamics of the bee/ant colony [61] and the aggregation of slime moulds itself is a response to other slime moulds trapped in unfavourable conditions [62]. Similarly, embryogenesis continues without a strictly causative impulse. One may argue that cells contain the information needed to form the organism, but the outcome itself is a result of interactions between genes and proteins at the sub-cellular and cells at the cellular level.

## AGENT-BASED AND CONTINUUM APPROACH

Another reason why agent-based models tend to do better than their continuum counterparts is because the latter tend to be population-based, relating observables to each other via equations that may either be algebraic, or capture variability temporally (ODE) or spatiotemporally (PDE) [66]. This is achieved by considering the impact of the system-level observables, disregarding the underlying cellular (individual) variation entirely. The homogeneity condition is essential for continuum models, as heterogeneity makes it impossible to obtain an analytical solution [67]. In doing so, these models overlook lower-level details as well as the augmentative impact of the (micro)environment heterogeneity on system evolution. Such an oversight cannot be accommodated while considering biological behaviour for, to argue in the cellular context, identical cells can generate non-identical colonies based on microenvironmental or intracellular cues [22,31]. Agent-based models allow one to study agent interactions and trace processes that emerge from such interactions. Such models are therefore better representational formalisms for biological systems and more accurate tools to deduce the effects of external stimuli, as they account for heterogeneity in responsiveness of individual cells—an integral constituent of most biological models.

It must, however, be stated that the agent-based approach and discrete computational approaches, in general, are typically less utilized to simulate bulk phenomena, where population behaviour holds more significance than individual behaviour, at least within currently available computational resources. So, while ABM can be used to model intercellular and cell–microenvironmental interactions, as suggested by Thorne *et al*. [33], the approach is not a pragmatic option for capturing gradients (chemical, electrical or energetic) that exist in the cells’ microenvironment—for which recourse to the more traditional PDE-based models is recommended. Integrating agent-based models with their continuum counterparts, as has been tried elsewhere [31,68–71], is an elegant and, from a biological perspective, a more precise way of addressing the non-trivial problem of modelling biological systems. Another weakness generally associated with ABM is the flexible and dynamic nature of agent interactions, which makes the patterns and outcomes of these interactions inherently unpredictable. This is a necessary evil associated with the agent-oriented approach, for it is precisely these flexible and dynamic interactions that enable agent-based models to capture emergent phenomena. Problems associated with the unpredictability, however, can be eliminated by conducting sensitivity [72] and parametric [73] analyses, and optimization procedures [9,32,74].

In terms of implementation, agent-based models are no different than their continuum counterparts. Allow us to make the case for the parallel. Simulating a physical process using transport phenomena—a continuum approach—requires identifying appropriate governing equations as well as boundary and initial conditions. This is followed by approximating the differential equations by a system of algebraic equations for variables at discrete locations in time and space: a process known as discretization. Numerical grids, which divide the geometry into finite sub-domains, act as the discrete locations where the aforementioned variables are solved. In the meshfree Smooth Particle Hydrodynamics [75]—an alternative continuum approach—instead of using static or moving partitions of the domain (meshes), it is covered by discrete elements known as particles, which act as locations where the aforementioned differential equations are solved [75,76]. Finally, the appropriate solution method is applied followed by setting the convergence criteria for the solution method [77].

Similarly, once the problem to be modelled via the ABM is identified, the appropriate set of hypotheses or logic/mathematical rules governing the agents must be developed. Ideally, these rules must have empirical relevance (and justification). Boundary and initial conditions regulating the model are also specified at this point [72]. The governing rules are implemented at each discrete agent location, at various discrete time steps, which are confined to the specified boundary (the environment). The choice of agents is quite important—after all, ‘you can’t model bulldozers with quarks’ [78]. Whether the agents are cell organelles, cells, tissues or organisms plays a significant role in determining the validity of the governing rules. The model is completed by choosing the time marching features physically relevant to the process being studied [72]. Finally, the model is calibrated and validated by comparing the computational data with its empirical analogue (and measuring the error function [72]). Refer to Figure 3 for the parallel between continuum and discrete approaches.

## AGENT-BASED MODELS

The level of detail embedded [72] in an agent-based model deserves a special mention. If the model is too simple, it might not prove fit enough to provide understanding and testable predictions; on the other hand, too complex a model will be computationally cumbersome [28]. As Grimm and Railsback [28] point out, there are two ways to fine-tune any such model: pattern-oriented modelling and method of strong inference [79]. Pattern-oriented modelling entails incorporation of details that allow emergence of empirically observed patterns—patterns being non-random events. This approach is, Grimm and Railsback [28] argue, parallel to the methodology applied in natural sciences, citing how the key to revealing the structure of DNA lay in the patterns that indicated internal organization. The pattern-oriented approach also makes the model testable at various hierarchical levels due to the underlying details linked to the system’s internal organization [28]. The strong inference approach involves developing and contrasting alternative theories—including null theories, which will not give rise to emergent properties—to determine the theory most amenable to reproducing the observed patterns [28]. The validated theories can then be used to model similar behaviours in other systems or environments [28].

Another point worthy of a special mention, we believe, in any review (or, for that matter, research article) covering agent-based models is the Science and Art of rule generation. Recourse to rules instead of (or in most cases, in addition to) mathematical equations, as suggested in this review previously, is the fundamental way in which the agent-based approach differs from its continuum counterparts. But, how can the user devise these rules? The iterative method—of constructing a rule (based on empirical facts), deploying it to computationally predict the empirical observation and optimizing it until statistical significance between the computational prediction and empirical observation is achieved—utilized is no different than the one used to develop the classical continuum models. Just as the creation of mathematical models begins with the simplest equation, which subsequently improves in complexity and level of detail, it is recommended that the initial set of rules designed are simple, followed by a gradual advancement in complexity and detail. However, to include or not to include a particular parameter: that is the ‘essential’ question [9,28,31,32,72,80,81] when it comes to ABM. Ideally, these parameters must originate from empirical data, and the system’s (significant) dependence on these parameters must be rigorously validated. This, unfortunately, is not always the case (or even possible) due, as suggested by Thorne *et al*. [32,80], to a lack of relevant published data, unpublished experiments and absence of more advanced protocols/apparatuses/techniques needed to conduct a particular experiment. In such cases, the iterative approach of rule-generation that leads to the prediction of observed global patterns is the most suitable option. The rule may (quite rightly) not be accepted until it is experimentally validated, but the availability of the rule (and the model) itself, in terms of offering alternative explanations, may in turn push for the experiment to become available.

However, in cases where data is available, and in certain cases—such as bioprocess or ‘omic data—where an overwhelmingly large amount of data is present, as Kaul *et al*. [31] suggest, recourse to statistical analyses and rule-mining paradigms is recommended. Broadly categorized as computational intelligence methods, the techniques are generally employed to construct predictive models based on available data [82,83]. However, as these models capture neither the heterogeneity of interactions between system components nor their governing physical laws, and, furthermore, are based on averaged global values, they tend to have limited predictive power. The techniques can, however, be used to extract (logical) rules from the available data [82,83]. The methodologies based on the underlying pattern recognition approach can be statistical, neural, evolutionary, genetic, tree-based or machine-learning-type [84]. Furthermore, based on whether rule-extraction relies on the presence of a class structure, the method itself can be classified as either supervised or unsupervised [84]. The basic idea of employing either of these techniques (especially in the case of Artificial Neural Networks) entails correlating data input with output via mathematical transformations, which, if applied to the input, will generate the output [84]. The mathematical transformations can be converted into logical rules [82], which can be subsequently employed to construct agent-based models.

Based on the rule-extraction approach utilized by the computational intelligence methods, they can be Naïve Bayesian, Artificial Neural Networks, Decision Trees or Support Vector Machines [85,86]. The abundance of rule-extracting methodologies necessitates a review of its own accord, as has been conducted in the following [82,85–87], perhaps of interest to the eager reader. Finally, the accuracy with which an agent-based model will predict and capture the global behaviours observed in a biological system heavily relies on the validity of the rules attributed to the system (in addition to the level of detail embedded in the model). Therefore, care must be taken in ensuring that post-extraction rule-refinement [87], pruning [88] and optimization [82] protocols are rigorously followed.

The use of agent-based models extends to the entire biological spectrum. The paradigm is especially useful to develop models based on ‘soft’ factors such as the irrational and subjective human behaviour employed to construct sociological models [24]. Agent-based models have been used in the field of cancer research [71,89–91], ecology [28,61,65], economics [92], immunology [9,34,55,56], tissue engineering [18,19,25,31,58,59,68–70,93,94] and clinical [23] and systems biology (the latter discussed in [95]). The most promising aspect of some of these models has been their ability to capture emergence [19,31,58,61,67,96].

### Standalone models

Reynolds’ Boids model [65], based on simple rules: agents avoiding collision, staying close to neighbours and matching the velocity of neighbours, leads to the emergent school-like aggregation of agents, and can be employed to model flocking behaviour of birds [9,28,61] or schooling of fishes [28,97]. On a similar note, Railsback and Harvey [98] developed an agent-based model to explain habitat selection patterns in stream salmonids, using input data and parameters representing cutthroat trout (*Oncorhynchus clarkii*). By contrasting three ‘theories’ (refer to the strong inference approach discussed in this section): maximizing current growth rate, current survival probability, or expected maturity, they reproduced numerically all habitat selection patterns (especially with the ‘maximizing the expected maturity’ rule). In the ecological context, the agent-based approach was utilized to simulate sea-lice (*Lepeophtheirus salmonis*) infestation patterns on a representative Atlantic salmon (*Salmo salar L*) population [99]. The basic motivation was to optimize Wrasse (which prey on the sea lice) densities so as to control the population of *L. salmonis* as the means of a pest management programme. An agent-based model developed by including a ‘threshold’ parameter into Thomas Schelling’s model of racial segregation [100] led to a rather robust emergent clustering behaviour that, despite being amenable to empirical verification, is practically quite challenging to validate due to unavailability of reliable and extensive data [67]. Furthermore, Mitchel Resnick’s Turtles, Termites, and Traffic Jams [61] review the various rule-sets utilized to model patterns observed in slime mould, ant and termite colonies. Resnick utilized the agent-based platform StarLogo.

Gary An [23] developed an agent-based model of innate immune response and used it to simulate clinical sepsis trials of anticytokine therapy, which produced patterns qualitatively similar to those published in the literature. Furthermore, An implemented a series of treatment regimens in the ABM to determine their impact on system mortality. No significant improvement was recorded. The investigation was meant to introduce ABM to clinicians, and the innate immune response model itself was able to successfully demonstrate counterintuitive system responses [23]. Segovia-Juarez *et al*. [101] used the agent-based approach to simulate the formation of granuloma in lungs post *Mycobacterium tuberculosis* infection. The alveolar lung tissue acted as the environment in which agents acted as ontologies for macrophages and T-cells. Model observations, such as the primary contribution of spatial distribution of T-cells (rather than the number of recruited T-cells) to a developing granuloma, have empirical basis. The investigators were the first to apply uncertainty and sensitivity analyses in this setting.

To determine how background peptides bound to Major Histocompatibility Complex (MHC) molecules—together referred to as pMHC—present on the surface of antigen-presenting cells influence antigen recognition by T-cells requires a computational model. This is due to the inherent physiological complexity of the process that relies on interactions between T-cells and their ligand on antigen-presenting cells or target cells (pMHC). In light of the extreme abundance of background peptides (compared with foreign ones), it seems intuitive to suggest that T-cells ignore the background proteins. An agent-based model developed by Casal *et al*. [56] suggests that T-cells rely instead on information gathered from all pMHC interactions and not just (selectively) from a few peptides. Along similar lines, employing an agent-oriented approach, Riggs *et al*. [55] developed a two-dimensional (2D) model of a lymph node that captured various empirically observed features of T-cell and dendritic cell dynamics. The model further suggests that a random search strategy, as opposed to chemotaxis (quite a counterintuitive thought), is more suited for a rare cognate T-cell to find its dendritic match, and thus activate T-cells. While supported by empirical evidence, the implications of this model need to be further investigated and validated.

In The Hallmarks of Cancer [102], Hanahan and Weinberg suggested six alterations in normal cell physiology that collectively lead to malignant growth. These included [102] self-sufficiency in growth signals, insensitivity to anti-growth signals, evasion of apoptosis, limitless replicative potential, sustained angiogenesis and tissue invasion and metastasis. Abbott *et al*. [89] developed CancerSim, an agent-based simulation, based on the Hanahan–Weinberg article [102], to simulate the dynamics through which cell populations acquire heterogeneity and the hallmarks of cancer. Mutation was introduced as a probabilistic parameter. Results from CancerSim were found to be in agreement with a(n) (ODE-based) continuum model that was also employed to model the hallmarks. Both, CancerSim and the continuum model suggested implicate cell death rather than genetic instability in driving the progression to cancer. This was in contrast to the pathways suggested by Hanahan and Weinberg, which place insensitivity to anti-growth signals at the beginning and limitless replication at the end of each pathway [89].

Lollini *et al*. [103] developed SimTriplex, an agent-oriented simulator, to investigate the minimum vaccination (Triplex vaccine) schedule that could afford immunological prevention of cancer in HER-2/neu transgenic mice at par with the currently implemented Chronic (administered for host’s lifetime) protocol. The experiments would have required a lot of experiments with associated cost implications. SimTriplex employs a minimal search strategy, which is based on a genetic algorithm, to describe the immune response activated by Triplex vaccine. Results from the investigation seem to suggest that the same efficacy as the Chronic protocol can be achieved by cutting down the number of vaccinations by roughly 40% [103]. Nagoski *et al*. [104] modelled the risk of contracting HIV, as a factor of heterogeneity of sexual motivation, in computer-generated artificial societies. A significant reason behind the use of ABM as a methodological approach was the opportunity to model systems where research data are either inaccessible or unethical. The fact that ABM can consider ‘soft’ variables such as stigma, discrimination, distrust of vaccines, etc. made it especially relevant as a modelling tool in this investigation. Gendered agents, hypothetical diseases and sexual motivation profiles constituted the three elements of the model. The model led to results such as high susceptibility to infection among the female-gendered agents earlier in their lives and emergence of ‘pockets of protection’ or infection-free zones that were not predicted but reflected patterns observed in human systems.

From a tissue engineering and regenerative medicine perspective, the agent-based approach has been utilized to model stem cell self-organization [58,59] in a niche [54], stem cell differentiation and division [58] and stem cell internal life cycle [19]. Furthermore, Galvao *et al*. [18] used a 2D agent-based computational model to investigate the role of stem cell therapy in tissue regeneration. They chose to model the chronic chagasic cardiomyopathy after bone marrow stem cell transplantation and therefore better understand the kinetics of cardiac tissue regeneration. The model could simulate apoptosis and differentiation and implicated concentration patterns of fibrotic regions and inflammatory cells (these categories corresponded to types of agents used in the model) as the most important factors in the kinetics of chronic chagasic cardiomyopathy regeneration after bone marrow stem cell transplantation. The results also attributed the reduction in fibrotic area to the initial fraction of bone marrow stem cells (another agent type in the model). Walker *et al*. [25,93] have been working on developing the model of epithelial tissue employing an agent-based model based on the social behaviour of cells. In the initial proof-of-concept model [93], they used rules explaining cellular behaviour such as cell cycle, bonding, spreading, migration and apoptosis to simulate growth characteristics of epithelial cells in monolayer culture under low and physiologic calcium ion (Ca^2+^) concentration. The computational results were found to qualitatively replicate the trends observed *in vitro*. An advanced form [25] of this model was used to describe the impact of extra-cellular calcium on the growth and differentiation of human keratinocytes. Furthermore, the model was used to invalidate the hypothesis that growth characteristics of the transformed HaCat (epithelial) cell line can be explained by simply ‘turning off’ the differentiation rule from the keratinocyte model, thereby demonstrating the application of agent-based models as hypothesis testing tools in biological investigations.

Thorne *et al*. [105] used the agent-based approach to describe cell-mediated changes in the geometry, composition and properties of an adapting vascular wall. The agents, representing endothelial and smooth muscle cells, displayed an array of behaviours such as proliferation, growth factor production, matrix production or degradation and apoptosis. The investigators utilized a ‘refined rule-set’ to model mouse aorta during homeostasis and in response to both transient and sustained increases in pressure. The agent-based model was compared with results derived from a pre-validated constrained mixture model of vascular adaptation at the tissue level. They concluded that their model was responsive to increased intramural wall shear stress in hypertension, but insensitive to transient elevations in the blood pressure. Long and Rekhi [106] used the agent-based approach to test the strategy that best governs cell movement during vessel regeneration as a function of vascular endothelial growth factors and brain-derived neurotrophic factor. The investigators compared the computational results with physical data derived by carrying out *in vitro* angiogenesis. Even though only three basic cellular behaviours—proliferation, migration and branching—were considered, the model was capable of predicting ‘growth for novel situations’. Certain aspects of the model, as the authors themselves acknowledged, were highly idealized. For example, the authors assumed spatiotemporal uniformity in the concentration of vascular endothelial growth factors and brain-derived neurotrophic factor, and focused the investigation to the first 24 hours of sprout growth.

Comparatively, Artel *et al*. [107] modelled sprouting angiogenesis in a porous scaffold using the agent-based approach. The objective of this investigation was to examine the impact of scaffold pore size on the rate of angiogenesis. The investigators relied on *in vivo* results to define the speed of vessel sprouting. The agents, representing capillary segments, were attributed behaviours such as elongation, branching and anastomosis. These behaviours were either stochastic or influenced by microenvironmental conditions. Results showed positive correlation between pore size and the rate of scaffold vascularization. Specifically, pore size between 160 and 270 µm was observed to support ‘rapid and extensive angiogenesis throughout the scaffold’.

### Multi-paradigm models

Being hierarchical in nature, most agent-based frameworks can be easily linked to various other computational paradigms [68]. This is perhaps the most desirable feature of the ABM paradigm, as it allows for the most precise representation of biological systems: the continuous environment encapsulating the autonomous agents. The so-called hybrid models [31,68–71] (among numerous others) use the agent-based approach to simulate biological interactions and decision-making, and partial/ordinary differential equations-based continuum approach to model the environment (gradients, concentrations, stresses, etc.)—features that would require enormous computational capabilities to be solved using the agent-based approach alone. Athale *et al*. [71] created a 2D multi-scale model of gene–protein interactions to simulate the decision-making approach that cancer cells employ to switch between proliferation and migration—cancer cells do not display the two phenotypes concurrently. The model included a novel intracellular module. They integrated the RePast toolkit (http://repast.sourceforge.net) with in-house-developed classes for representing molecules, reactions and sub-cellular compartments. The evolution of variables, such as molecular concentrations, is represented using ordinary differential equations and cellular behaviour using the agent-based framework. The system can simulate tumour growth over several orders of magnitude. Bailey *et al*. [70] developed a model underpinned by a blood flow network simulation to dynamically track inflammatory cell navigation through microvasculature to a simulated skeletal muscle capillary bed via interactions with the endothelium. The microvascular network was derived from mouse spinotrapezius muscle, and combined with a network flow model designed to calculate haemodynamic parameters (such as fluid flow and wall shear stress) throughout the simulated microvascular network. The investigation yielded results consistent with literature data, including monocyte migration occurring primarily in the venules (even though differences in endothelial cell phenotype were not explicitly accounted for in the model) and low dependence of monocytes on selectins for firm adhesion (a non-intuitive result) [70]. The network flow model was implemented in MATLAB, whereas the agent-based model in NetLogo.

Adra *et al*. [68] integrated Flexible Large-scale Agent-based Modelling Environment (FLAME) with COmplex PAthway SImulator and a physical numerical solver [93] to develop a three-dimensional (3D) multi-scale model to grow a virtual piece of epidermis from a collection of stem cells and derive a set of biological rules for transforming growth factor-beta 1 (TGF-β1; cytokine) during epidermal wound healing. In this investigation, the agent-based model was used to capture biological rules governing intercellular interactions in the human epidermis; COmplex PAthway SImulator was used to simulate the expression and signalling of TGF-β1 at the intracellular level and the physical solver was used at the continual level to resolve forces exerted between cells. The model was able to successfully simulate many described keratinocyte behaviours and TGF-β1 intracellular mechanisms. Sun *et al*. [69] utilized the same approach to develop a 3D multi-scale model of the formation of skin epithelium based on rule-sets involving TGF-β1 to test the role TGF-β1 plays in wound healing. Wounds were introduced into the model, which was then used to observe keratinocyte behaviour during healing and explore various hypotheses concerning the role of TGF-β1 by manipulating the rule-set associated with the cytokine. The model supported the *in vivo/in vitro* observation that TGF-β1 maintains a balance between keratinocyte proliferation and differentiation during wound healing, and further indicated that disruption of TGF-β1 expression or signalling could impact the healing process.

Solovyev *et al*. [108] constructed a hybrid model of ischaemia-induced hyperemia (sudden increase in skin blood flow following ischaemia) and pressure ulcer formation by combining an ODE model of blood flow and reactive hyperemia, and ABM of skin injury, inflammation and ulcer formation. Their primary objective was to gain useful insights into post-spinal cord injury (SCI) pressure ulcers, which may result from prolonged tissue ischaemia. The agent-based aspect of the model simulated injury, inflammation and ulcer formation by capturing interactions between oxygen, pro-inflammatory elements, anti-inflammatory elements and skin damage (agents used in the model). Experimental data from human subjects (six SCI patients and six non-injured subjects) were used to calibrate the ODEs used in the model. The model suggested a higher propensity for ulceration in the patients compared with the subjects. Despite certain limitations identified by the authors themselves, the model can be employed as a diagnostic platform for post-SCI ulcer formation.

**Figure 4:**
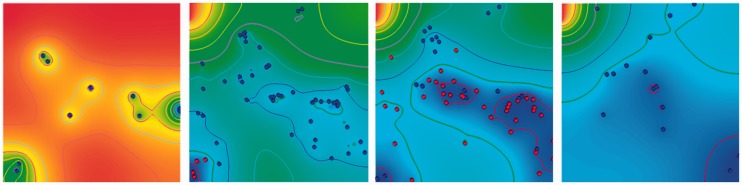
This sequence displays results generated from a platform developed by integrating the agent-based with the continuum approach. The figure shows various stages of cell chemotaxis under the influence of an arbitrary chemokine. The cells, on sensing chemokine-deficient conditions, try to move into chemokine-rich regions. The four frames were captured at 0, 20, 30 and 50 hours of experiment time. Whereas chemokine concentration in the cellular microenvironment was modelled using the transport phenomena solver, cellular chemotaxis was simulated using FLAME. The image first appeared in [31] and was reprinted under the Creative Commons Attribution License. A colour version of this figure is available at BIB online: http://bib.oxfordjournals.org.

Kaul *et al*. [31] integrated FLAME with a multi-physics transport phenomena platform (CFD-ACE+, ESI Group, Paris, France) to capture dynamic reciprocity [109] in a 3D bioreactor. Through the model, the authors investigated the impact of system initial and boundary conditions on its overall evolution. The platform capable of supporting 2D (refer to Figure 4) and 3D models can simulate most cellular behaviours and captured not only the differences in system evolution due to the differing initial/boundary conditions but the similarities as well. Furthermore, as the cells tried to evade hypoxic regions inside the bioreactor, they aggregated around regions with threshold oxygen concentration in a manner that can only be described as emergent (although this needs experimental verification). The proof-of-concept demonstrated the utility of such a platform as a hypothesis testing (experimental purposes) and design optimization (commercial purposes, as during bioreactor concept selection phase) tool.

Due to the nature of this document, we were unable to delve more critically into the scope, design, functionality and results of the agent-based models presented here and therefore urge the reader to explore the articles relevant to them. We also hope that investigators whose seminal and relevant work in this field we were unable to cite will forgive the nature of this review, primarily aimed to evaluate the merit of agent-oriented approach in simulating biological phenomena, and associated space constraints.

## CONCLUSIONS

The versatility of the agent-based approach makes it a particularly appealing modelling framework to analyse complex systems. Despite its successes and enormous potential, the agent-based approach remains nascent and requires utilization on a larger scale, not only by biologists, due to its intuitive and simple execution, but also by mathematicians and engineers alike, as either a standalone approach or coupled with other modelling paradigms. An agent-oriented view might not only be able to explain biological behaviour, but uncover the rules leading to emergent behaviour in such systems and the macroscopic dynamics that regulate them. We conclude this article by reiterating the features—a summation of the literature reviewed to write this article—that any modelling approach required to simulate biological complexity must possess. These include the ability of the approach to simulate non-linear and dynamic behaviour, synthesize relevant ‘constituent-constituent’ and ‘constituent-environment’ interactions, track the evolution of various constituents that are heterogeneous in nature, develop memory of various prior constituent interactions, adapt to the external environment and permit visualization of emergent phenomena that will result from the combined interactions of system constituents. The agent-based paradigm is probably the most perfect embodiment of these characteristics.

**Key Points**

- Agent-based models allow the study of component-level interactions and trace processes that emerge from such interactions. Such models are therefore better representational formalisms for biological systems and more accurate tools to deduce the effects of external stimuli, as they account for heterogeneity in responsiveness of individual cells—an integral constituent of most biological models.
- ABM ‘discretizes’ the system being modelled into a collection of autonomous decision-making entities that act at each of several discrete time steps based on their local information and rule-set attributed to them.
- Just as the creation of continuum models begins with the simplest equation, which subsequently improves in complexity and level of detail, it is recommended that the initial set of rules designed are simple, followed by a gradual advancement in complexity and detail.
- The decentralized manner of targeting complex systems is perhaps the strongest argument in favour of using ABM to simulate biological processes. Unlike the continuum approach with a distinct cause-and-effect motif, the agent-based approach relies on interactions—among agents coupled with their environment—to capture and explain macroscopic observables.

## FUNDING

Himanshu Kaul gratefully acknowledges support through a Department of Engineering Science, University of Oxford, Scholarship.
